# Supplementary material for: Trends in Cancer Incidence and Mortality in US Adolescents and Young Adults, 2016–2021
Source: Cancers (Basel). 2024 Sep 14;16(18):3153. doi: 10.3390/cancers16183153 (PMC11430075; doi:10.3390/cancers16183153)
Supplement: Supplementary file 1 [file cancers-16-03153-s001.zip › cancers-3188999-supplementary.pdf]

Trends in Cancer Incidence and Mortality in US Adolescents and Young Adults, 2016-2021, Li Zhang, Online Supplement Document

Table S1. AYA cancer incidence and mortality by sex

	Incidence						Mortality					
**Cancer Subtype**	**Male (n=88,884)**			**Female (n=141,091)**			**Male (n=14,533)**			**Female (n=14,867)**		
	Rate	Count	%	Rate	Count	%	Rate	Count	%	Rate	Count	%
**1.Leukemias**	5.0	8226	9.3	4.2	6635	4.7	1.4	1979	13.6	0.9	1274	8.6
1.1 Acute lymphoid leukemia	1.4	2399		0.8	1264		0.6	854		0.3	446	
1.2 Acute myeloid leukemia	1.1	1901		1.2	1940		0.4	625		0.4	556	
1.3 Chronic myeloid leukemia	0.9	1432		0.6	948		0.1	176		0.1	75	
1.4 Other and unspecified leukemia	0.5	740		0.4	548		0.1	128		0.1	76	
**2.Lymphomas**	8.2	13626	15.3	7.0	11195	7.9	1.1	1604	11.0	0.6	869	5.9
2.1 non-Hodgkin lymphoma	4.1	6745		3.1	4939		0.7	1002		0.4	511	
2.2 Hodgkin lymphoma	3.4	5701		3.3	5430		0.3	420		0.2	247	
**3.CNS and Other Intracranial and Intraspinal Neoplasms**	3.0	4975	5.6	2.3	3702	2.6	1.2	1753	12.0	0.8	1134	7.6
3.1 Astrocytoma (Astroglia and related neoplasms)	2.7	4492		2.1	3364		1.1	1527		0.7	997	
3.1.1 Oligodendrogliomas	0.4	691		0.3	527		0.1	187		0.1	150	
3.1.2 Glioblastoma (invasive)	0.6	1051		0.4	622		0.4	559		0.2	304	
3.1.3 Ependymoma (invasive)	0.2	283		0.2	278		0.0	57		0.0	46	
3.1.4 Other astrocytoma/astroglial neoplasms	1.5	2467		1.2	1937		0.5	724		0.4	497	
3.2 Medulloblastoma	0.2	267		0.1	146		0.1	140		0.1	73	
**4. Sarcomas**	3.2	5272	5.9	3.0	4795	3.4	1.1	1548	10.7	0.8	1122	7.5
4.1 Osteosarcoma	0.4	726		0.3	461		0.2	341		0.2	220	
4.2 Chondrosarcoma	0.2	319		0.2	290		0.0	41		0.0	30	
4.3 Ewing tumor	0.3	582		0.2	362		0.2	267		0.1	170	
4.3.1 bone tumors	0.2	376		0.1	191		0.1	181		0.1	107	
4.3.2 Soft tissue sarcomas	0.1	206		0.1	171		0.1	86		0.0	63	
4.4 Fibromatous neoplasms	0.5	868		0.6	890		0.0	42		0.0	32	
4.5 Liposarcoma	0.3	497		0.2	350		0.0	65		0.0	36	
4.6 Synovial sarcoma	0.2	304		0.2	334		0.1	131		0.1	90	
4.7 Leiomyosarcoma	0.1	175		0.3	401		0.0	31		0.1	102	
4.8 Rhabdomyosarcoma	0.2	326		0.2	245		0.1	195		0.1	110	
4.9 Gastrointestinal stromal tumor	0.2	290		0.2	285		0.0	24		0.0	14	
4.10 Other soft tissue sarcoma	0.3	485		0.4	585		0.1	174		0.1	190	
4.11 Other bone tumors	0.1	141		0.1	131		0.0	24		0.0	18	
**5. Blood and lymphatic vessel tumors**	0.9	1476	1.7	0.2	262	0.2	0.3	489	3.4	0.1	72	0.5
5.1.1 Specified (non-Kaposi sarcoma)	0.1	221		0.1	225		0.1	79		0.0	63	
5.1.2 Kaposi sarcoma	0.8	1255		0.0	37		0.3	410		0.0	9	
**6. Nerve sheath tumors (malignant)**	0.2	256	0.3	0.1	219	0.2	0.1	124	0.9	0.1	89	0.6
**7. Gonadal and related tumors**	11.5	19471	21.9	3.0	4764	3.4	0.9	1262	8.7	0.5	681	4.6
7.1 Testis	11.0	18668		-	-		0.7	1028		-	-	
7.2 Ovary	-	-		2.8	4322		-	-		0.5	622	
7.3 Germ cell and trophoblastic (GCT)-CNS	0.2	317		0.0	46		0.0	57		0.0	13	
7.4 GCT (excluding CNS, testis, ovary)	0.3	486		0.2	388		0.1	177		0.0	44	

**8. Melanoma and Skin Carcinomas**	3.6	5773	6.5	6.8	10642	7.5	0.3	408	2.8	0.3	341	2.3
**9. Carcinomas**	18.2	28850	32.5	63.1	97110	68.8	3.6	4998	34.4	6.7	8849	59.5
9.1 Thyroid carcinoma	4.1	6720		17.9	28454		0.1	124		0.2	232	
9.2 Other carcinoma of head and neck	1.6	2563		1.3	2062		0.3	446		0.2	207	
9.2.1 Nasopharyngeal carcinoma	0.2	384		0.1	219		0.1	77		0.0	31	
9.2.2 Lip, oral cavity and pharynx	0.9	1345		0.6	939		0.2	237		0.1	122	
9.2.3 Salivary gland	0.3	485		0.4	694		0.0	54		0.0	22	
9.2.4 Other carcinoma of head and neck	0.2	349		0.1	210		0.1	78		0.0	32	
9.3 Carcinoma of gastrointestinal tract	7.4	11742		7.6	11639		2.3	3221		1.8	2415	
9.3.1 Carcinoma of esophagus	0.2	352		0.1	93		0.1	171		0.0	41	
9.3.2 Carcinoma of stomach	0.7	1179		0.8	1207		0.4	609		0.4	520	
9.3.3 Carcinoma of small intestine	0.2	380		0.3	414		0.0	63		0.0	48	
9.3.4 Carcinoma of colon	3.1	4882		3.5	5466		0.6	880		0.6	785	
9.3.5 Carcinoma of rectum	1.7	2675		1.5	2286		0.4	570		0.3	395	
9.3.6 Carcinoma of anus	0.2	337		0.1	215		0.1	73		0.0	34	
9.3.7 Carcinoma of liver and intrahepatic bile duct	0.5	832		0.4	564		0.3	430		0.2	239	
9.3.8 Carcinoma of gallbladder and other extrahepatic biliary	0.1	207		0.1	199		0.1	81		0.1	79	
9.3.9 Carcinoma of Pancreas	0.5	837		0.7	1106		0.2	306		0.2	226	
9.4 Carcinoma of lung, bronchus, and trachea	0.8	1212		0.9	1366		0.3	460		0.3	368	
9.5 Carcinoma of skin	0.1	132		0.1	130		0.0	12		0.0	6	
9.6 Carcinoma of breast	0.1	135		22.3	33584		0.0	12		2.5	3222	
9.7 Carcinoma of genital sites (non ovary or testis)	0.2	318		10.2	15628		0.0	31		1.4	1822	
9.7.1 Carcinoma of uterine cervix	-	-		6.1	9373		-	-		1.1	1422	
9.8 Carcinoma of urinary tract	3.4	5362		2.3	3468		0.3	433		0.2	277	
9.8.1 Carcinoma of kidney	2.7	4136		1.9	2914		0.3	346		0.1	197	
9.8.2 Carcinoma of bladder	0.7	1169		0.3	522		0.1	73		0.1	71	
9.9 Carcinoma of other and ill-defined sites	0.4	666		0.5	779		0.2	259		0.2	300	
**10. Miscellaneous specified neoplasms**	0.2	307	0.3	0.4	570	0.4	0.1	132	0.9	0.1	122	0.8
**11. Unspecified malignant neoplasms, except CNS**	0.4	652	0.7	0.8	1197	0.9	0.2	236	1.6	0.2	314	2.1
**Race/ethnicity**												
Non-Hispanic White	63.1	48933	55.0	102.3	75231	53.3	9.7	6259	43.1	10.4	6369	42.9
Non-Hispanic Black	**43.0***	8587	9.7	**78.3***	15783	11.2	**13.6***	2351	16.2	**15.1***	2631	17.7
Non-Hispanic American Indian/Asian pacific	**41.4***	7212	8.1	**77.4***	14120	10.0	**8.3***	1310	9.0	**8.6***	1419	9.5
Hispanic (All Races)	**49.7***	24152	27.2	**83.5***	35957	25.5	**10.4***	4613	31.7	**11.3***	4448	29.9
**Metropolitan status**												
Metropolitan	54.2	81078	91.2	90.2	128831	91.3	10.1	13093	90.1	10.9	13405	90.2
Non-Metropolitan	55.5	7716	8.7	**98.8***	12116	8.6	**11.5***	1411	9.7	**13.3***	1434	9.6

Unknown/missing (Alaska or Hawaii)	60.8	90	0.1	**115.2***	144	0.1	**20.2***	29	0.2	**22.4***	28	0.2

All incidence and mortality rates were age-adjusted and reported per 100,000 persons.
SEER*Stat reports AYAs with missing/unknown metropolitan/nonmetropolitan status from Alaska or Hawaii.

Table S2. Trends in cancer incidence and mortality for males and females, 2016-2021

Cancer Subtype	Incidence				Mortality			
	Male		Female		Male		Female	
	APC (95% CI)	P value	APC (95% CI)	P value	APC (95% CI)	P value	APC (95% CI)	P value
**1.Leukemias**	-0.3 (-3.6, 3.2)	0.85	0.03 (-2.3, 2.4)	0.98	**-1.4 (-2.3, -0.6)**	**0.01**	**-2.6 (-4.7, -0.5)**	**0.03**
1.1 Acute lymphoid leukemia	1.1 (-2.6, 4.9)	0.46	**3.4 (1.6, 5.2)**	**0.01**	-2.1 (-5.6, 1.6)	0.19	-0.4 (-3.3, 2.6)	0.72
1.2 Acute myeloid leukemia	0.2 (-7.1, 8.0)	0.96	-0.5 (-6.5, 5.9)	0.85	-2.8 (-6.6, 1.3)	0.13	-4.0 (-10.6, 3.2)	0.19
1.3 Chronic myeloid leukemia	-2.3 (-6.7, 2.2)	0.22	-0.3 (-4.3, 3.8)	0.83	-1.9 (-13.7, 11.5)	0.7	-5.2 (-23.0, 16.7)	0.51
1.4 Other and unspecified leukemia	1.6 (-5.1, 8.8)	0.558	-0.5 (-9.0, 8.7)	0.88	**11.9 (5.2, 19.1)**	**0.01**	2.1 (-21.1, 32.0)	0.84
**2.Lymphomas**	-1.7 (-3.9, 0.5)	0.09	**-1.6 (-2.9, -0.2)**	**0.03**	-3.2 (-6.7, 0.4)	0.07	1.3 (-4.3, 7.2)	0.57
2.1 non-Hodgkin lymphoma	**-2.8 (-4.1, -1.5)**	**0.004**	-2.4 (-4.8, 0.1)	0.06	**-5.9 (-10.6, -0.9)**	**0.03**	0.5 (-3.9, 5.1)	0.75
2.2 Hodgkin lymphoma	-0.2 (-3.7, 3.4)	0.86	-0.4 (-2.9, 2.1)	0.66	5.1 (-2.0, 12.7)	0.12	4.1 (-9.9, 20.3)	0.48
**3.CNS and Other Intracranial and Intraspinal Neoplasms (All behavior)**	-1.4 (-2.7, 0.1)	0.06	-1.4 (-3.2, 0.5)	0.11	1.2 (-2.5, 5.0)	0.44	-2.4 (-8.1, 3.6)	0.32
3.1 Astrocytoma (Astroglia and related neoplasms)	-1.2 (-2.8, 0.4)	0.10	-1.2 (-3.6, 1.4)	0.27	1.3 (-2.4, 5.1)	0.40	-2.1 (-8.1, 4.2)	0.39
3.1.1 Oligodendrogliomas	0.8 (-2.4, 4.0)	0.55	-0.2 (-8.0, 8.3)	0.96	-1.0 (-11.7, 10.9)	0.81	2.5 (-7.6, 13.7)	0.54
3.1.2 Glioblastoma (invasive)	-1.9 (-10.0, 6.9)	0.57	-5.0 (-14.7, 5.8)	0.26	2.1 (-1.4, 5.6)	0.17	-5.1 (-15.0, 6.0)	0.26
3.1.3 Ependymoma (invasive)	-4.9 (-11.1, 1.8)	0.11	**-5.9 (-10.8, -0.8)**	**0.03**	6.5 (-8.9, 24.4)	0.33	-3.5 (-20.2, 16.8)	0.63
3.1.4 Other astrocytoma/astroglial neoplasms	-0.9 (-4.5, 2.9)	0.56	0.6 (-3.1, 4.3)	0.70	1.1 (-7.6, 10.5)	0.76	-1.5 (-6.3, 3.5)	0.45
3.2 Medulloblastoma	-2.9 (-9.0, 3.6)	0.28	-1.5 (-11.6, 9.7)	0.71	3.8 (-6.2, 14.9)	0.36	-0.9 (-7.9, 6.5)	0.74
**4. Sarcomas**	-1.9 (-4.5, 0.8)	0.12	-3.3 (-7.5, 1.1)	0.11	0.02 (-1.3, 1.4)	0.97	-0.2 (-6.1, 6.1)	0.92
4.1 Osteosarcoma	-1.3 (-3.9, 1.5)	0.27	-3.0 (-7.5, 1.1)	0.43	-1.2 (-9.9, 8.3)	0.73	-1.2 (-8.4, 6.5)	0.67
4.2 Chondrosarcoma	2.7 (-9.4, 16.4)	0.59	-5.6 (-14.2, 4.0)	0.18	6.1 (-10.6, 25.8)	0.39	-0.1 (-26.0, 34.8)	0.99
4.3 Ewing tumor	-1.5 (-7.6, 5.0)	0.54	-2.5 (-14.5, 11.2)	0.62	-1.1 (-7.1, 5.3)	0.66	0.6 (-19.7, 26.0)	0.95
4.3.1 bone tumors	-0.9 (-8.3, 7.0)	0.75	-1.2 (-9.2, 7.4)	0.71	-3.7 (-9.5, 2.4)	0.16	-1.6 (-15.0, 14.0)	0.78
4.3.2 Soft tissue sarcomas	-2.9	0.50	-3.4	0.66	5.4	0.45	4.7	0.75

	(-13.1, 8.4)		(-21.1, 18.2)		(-11.5, 25.6)		(-28.4, 53.1)	
4.4 Fibromatous neoplasms	**-6.8 (-12.8, -0.4)**	**0.04**	-9.1 (-23.3, 7.8)	0.20	-8.3 (-34.9, 29.1)	0.52	-4.0 (-28.5, 28.7)	0.72
4.5 Liposarcoma	-3.0 (-10.4, 5.0)	0.34	-5.7 (-13.6, 2.9)	0.14	3.3 (-10.5, 19.3)	0.56	-5.5 (-33.4, 34.2)	0.68
4.6 Synovial sarcoma	-1.7 (-9.1, 6.4)	0.59	**-4.4 (-8.5, -0.1)**	**0.04**	-0.7 (-15.9, 17.1)	0.91	2.8 (-12.2, 20.3)	0.65
4.7 Leiomyosarcoma	-11.0 (-21.4, 0.8)	0.06	-2.7 (-13.0, 8.8)	0.53	-13.0 (-40.8, 28.0)	0.37	-6.7 (-16.1, 3.7)	0.14
4.8 Rhabdomyosarcoma	-1.5 (-10.8, 8.7)	0.69	5.7 (-2.7, 14.9)	0.13	3.2 (-10.6, 19.1)	0.58	8.0 (-5.7, 23.7)	0.19
4.9 Gastrointestinal stromal tumor	8.2 (-11.8, 32.7)	0.34	11.7 (-2.0, 27.4)	0.08	-6.6 (-25.6, 17.3)	0.45	14.0 (-25.6, 74.6)	0.44
4.10 Other soft tissue sarcoma	-1.8 (-6.1, 2.8)	0.33	-1.6 (-10.0, 7.6)	0.64	3.6 (-1.6, 9.1)	0.13	3.4 (-6.0, 13.7)	0.38
4.11 Other bone tumors	-0.6 (-12.9, 13.3)	0.90	-2.7 (-10.7, 6.0)	0.43	12.9 (-4.8, 33.9)	0.12	8.7 (-12.7, 35.4)	0.35
**5. Blood and lymphatic vessel tumors**	-0.9 (-8.7, 7.6)	0.79	**7.7 (5.8, 9.6)**	**0.003**	-3.9 (-9.8, 2.4)	0.15	19.7 (-11.4, 61.7)	0.17
5.1.1 Specified (non- Kaposi sarcoma)	**9.3 (0.6, 18.6)**	**0.04**	7.2 (-0.8, 15.8)	0.07	3.8 (-6.0, 14.6)	0.36	21.2 (-7.1, 58.1)	0.12
5.1.2 Kaposi sarcoma	-2.5 (-10.6, 6.4)	0.46	2.8 (-34.6, 61.5)	0.87	-5.2 (-13.3, 3.7)	0.17	-	-
**6. Nerve sheath tumors(malignant)**	1.8 (-8.8, 13.6)	0.68	1.7 (-11.3, 16.7)	0.75	**15.4 (0.01, 33.3)**	**0.047**	7.4 (-20.1, 44.3)	0.54
**7. Gonadal and related tumors**	-0.9 (-2.1, 0.3)	0.10	0.8 (-0.7, 2.3)	0.22	3.9 (-1.1, 9.2)	0.1	0.2 (-6.6, 7.4)	0.95
7.1 Testis	-0.9 (-2.2, 0.5)	0.14	-	-	5.1 (-1.2, 11.8)	0.09	-	-
7.2 Ovary	-	-	1.4 (-0.2, 3.1)	0.07	-	-	-0.2 (-7.2, 7.4)	0.95
7.3 Germ cell and trophoblastic (GCT)-CNS	0.6 (-5.1, 6.6)	0.80	-1.8 (-22.2, 24.0)	0.84	0.8 (-23.0, 31.8)	0.94	-	-
7.4 GCT (excluding CNS, testis, ovary)	-3.6 (-13.4, 7.4)	0.40	**-5.8 (-8.7, -2.8)**	**0.01**	-1.0 (-11.5, 10.8)	0.82	-1.5 (-13.3, 11.9)	0.76
**8. Melanoma and Skin Carcinomas**	**-6.4 (-10.2, -2.5)**	**0.01**	**-4.3 (-7.7, -0.9)**	**0.032**	**-7.5 (-12.6, -2.2)**	**0.02**	-7.1 (-17.8, 5.0)	0.17
**9. Carcinomas**	-0.5 (-2.5, 1.5)	0.51	-0.7 (-3.1, 1.8)	0.48	0.2 (-1.8, 2.2)	0.80	-0.7 (-1.9, 0.5)	0.16
9.1 Thyroid carcinoma	-1.9 (-4.6, 0.8)	0.12	-2.5 (-6.7, 1.9)	0.18	-6.0 (-16.0, 5.2)	0.20	6.7 (-1.1, 15.2)	0.08
9.2 Other carcinoma of head and neck	-2.9 (-6.1, 0.4)	0.07	-1.6 (-5.8, 2.7)	0.35	0.9 (-5.7, 8.0)	0.73	0.8 (-15.0, 19.4)	0.90
9.2.1 Nasopharyngeal carcinoma	-1.2 (-7.6, 5.7)	0.65	1.7 (-6.0, 10.1)	0.58	-7.9 (-23.6, 10.9)	0.29	-	-
9.2.2 Lip, oral cavity and pharynx	-3.5 (-7.6, 0.7)	0.08	-2.3 (-9.2, 5.2)	0.44	1.7 (-12.3, 17.9)	0.77	0.2 (-13.1, 15.3)	0.98
9.2.3 Salivary gland	-2.6 (-7.2, 2.3)	0.21	-1.4 (-7.7, 5.4)	0.60	11.0 (-5.7, 30.6)	0.15	-1.3 (-42.3, 68.8)	0.95
9.2.4 Other carcinoma of head and neck	-3.2 (-15.6, 11.1)	0.55	-2.3 (-11.3, 7.5)	0.53	2.4 (-14.5, 22.5)	0.74	-9.8 (-31.6, 19.0)	0.36
9.3 Carcinoma of gastrointestinal tract	0.8 (-1.2, 2.8)	0.33	1.3 (-0.7, 3.3)	0.15	0.8 (-2.1, 3.9)	0.48	-0.5 (-4.3, 3.5)	0.75

9.3.1 Carcinoma of esophagus	1.6 (-10.2, 14.9)	0.74	7.5 (-4.9, 21.6)	0.18	7.8 (-5.6, 23.2)	0.19	-0.7 (-21.9, 26.2)	0.94
9.3.2 Carcinoma of stomach	-3.4 (-7.2, 0.6)	0.08	-0.6 (-3.3, 2.2)	0.59	-1.8 (-9.0, 6.0)	0.55	0.2 (-2.9, 3.4)	0.84
9.3.3 Carcinoma of small intestine	2.2 (-6.0, 11.1)	0.51	2.3 (-4.7, 9.9)	0.42	-9.3 (-18.6, 1.2)	0.07	3.6 (-17.7, 30.5)	0.69
9.3.4 Carcinoma of colon	0.6 (-1.8, 3.1)	0.52	1.1 (-2.4, 4.7)	0.45	2.5 (-1.6, 6.8)	0.16	0.4 (-7.0, 8.3)	0.91
9.3.5 Carcinoma of rectum	2.3 (-1.7, 6.5)	0.19	3.2 (-0.2, 6.7)	0.06	2.8 (-4.7, 10.9)	0.37	-1.7 (-9.9, 7.2)	0.61
9.3.6 Carcinoma of anus	5.5 (-1.5, 12.9)	0.09	-1.0 (-17.4, 18.7)	0.89	2.2 (-6.2, 11.4)	0.52	-	-
9.3.7 Carcinoma of liver and intrahepatic bile ducts	-1.9 (-9.4, 6.2)	0.54	-0.4 (-7.8, 7.5)	0.88	-2.9 (-8.9, 3.6)	0.28	-6.2 (-12.6, 0.7)	0.07
9.3.8 Carcinoma of gallbladder and other extrahepatic biliary	-1.8 (-12.1, 9.7)	0.67	3.7 (-7.3, 16.1)	0.42	6.6 (-14.0, 32.2)	0.45	-8.0 (-20.5, 6.4)	0.19
9.3.9 Carcinoma of Pancreas	3.8 (-4.8, 13.2)	0.30	1.1 (-5.4, 7.9)	0.68	0.7 (-5.9, 7.7)	0.80	5.0 (-5.0, 16.0)	0.25
9.4 Carcinoma of lung, bronchus, and trachea	-3.1 (-6.3, 0.2)	0.06	-5.7 (-11.2, 0.1)	0.05	-4.0 (-12.1, 4.9)	0.27	-1.5 (-7.5, 4.8)	0.52
9.5 Carcinoma of skin	-6.5 (-23.4, 14.2)	0.40	-2.5 (-20.0, 19.0)	0.74	-	-	-	-
9.6 Carcinoma of breast	6.7 (-7.6, 23.3)	0.28	0.9 (-1.0, 2.8)	0.28	-2.2 (-35.6, 48.5)	0.89	-1.6 (-5.0, 1.8)	0.25
9.7 Carcinoma of genital sites (non ovary or testis)	4.7 (-11.9, 24.4)	0.51	-1.5 (-4.4, 1.5)	0.23	1.8 (-24.4, 37.1)	0.88	0.1 (-5.1, 5.6)	0.96
9.7.1 Carcinoma of uterine cervix	-	-	-3.0 (-6.4, 0.5)	0.07	-	-	-2.1 (-7.3, 3.4)	0.34
9.8 Carcinoma of urinary tract	-0.7 (-5.0, 3.9)	0.70	-2.3 (-5.8, 1.3)	0.15	-1.9 (-6.6, 3.1)	0.35	-6.2 (-14.4, 2.8)	0.12
9.8.1 Carcinoma of kidney	-0.3 (-4.9, 4.6)	0.87	-1.3 (-4.5, 2.0)	0.32	**-4.5 (-8.1, -0.7)**	**0.03**	-8.8 (-20.0, 4.0)	0.12
9.8.2 Carcinoma of bladder	-2.0 (-8.3, 4.6)	0.43	-6.5 (-13.0, 0.3)	0.06	2.8 (-17.2, 27.6)	0.74	3.9 (-10.5, 20.5)	0.52
9.9 Carcinoma of other and ill-defined sites, NOS	3.4 (-2.2, 9.3)	0.17	4.9 (-1.0, 11.2)	0.08	3.4 (-3.5, 10.7)	0.25	0.5 (-9.5, 11.6)	0.90
**10. Miscellaneous specified neoplasms**	4.9 (-9.2, 21.2)	0.41	7.4 (-6.0, 22.6)	0.21	7.6 (-5.8, 22.8)	0.20	-1.5 (-14.6, 13.6)	0.78
**11. Unspecified Malignant Neoplasms, except CNS**	-0.03 (-3.7, 3.7)	0.98	**-6.5 (-11.4, -1.4)**	**0.03**	7.5 (-3.1, 19.3)	0.12	1.1 (-5.2, 7.7)	0.67
Any cancer type, Total	-1.3 (-3.0, 0.5)	0.11	-1.1 (-3.2, 1.1)	0.25	-0.1 (-0.6, 0.5)	0.75	**-0.8 (-1.4, -0.2)**	**0.02**

APC: annual percentage change.

Table S3. AYA cancer incidence rates (age at diagnosis, 15-39 y), and mortality rates for death from cancer (age at death, 15-39 y) from SEER-22, 2016-2021.

Cancer Subtype	Incidence			Mortality		
	Rate	Count	%	Rate	Count	%
**1.Leukemias**	**4.7**	15166	6.5	**1.3**	3539	11.3
1.1 Acute lymphoid leukemia	**1.1**	3713		0.5	1339	
1.2 Acute myeloid leukemia	**1.2**	4014		0.4	1383	

1.3 Chronic myeloid leukemia	0.8	2401		0.1	256	
1.4 Other and unspecified leukemia	0.4	1306		0.1	218	
**2.Lymphomas**	**7.7**	25130	10.7	0.9	2609	8.4
2.1 non-Hodgkin lymphoma	**3.7**	11878		0.6	1604	
2.2 Hodgkin lymphoma	**3.4**	11184		0.2	677	
**3.CNS and Other Intracranial and Intraspinal Neoplasms**	**2.7**	8793	3.7	1.1	3047	9.8
3.1 Astrocytoma (Astroglia and related neoplasms)	**2.5**	7967		1.0	2671	
3.1.1 Oligodendrogliomas	0.4	1230		0.1	356	
3.1.2 Glioblastoma (invasive)	0.5	1732		0.3	947	
3.1.3 Ependymoma (invasive)	0.2	567		0.04	108	
3.1.4 Other astrocytoma/astroglial neoplasms	**1.4**	4438		0.5	1260	
3.2 Medulloblastoma	0.1	417		0.1	218	
**4. Sarcomas**	3.2	10249	4.4	1.0	2818	9.0
4.1 Osteosarcoma	0.4	1200		0.2	603	
4.2 Chondrosarcoma	0.2	616		0.03	75	
4.3 Ewing tumor	0.3	968		0.2	453	
4.3.1 bone tumors	0.2	579		0.1	293	
4.3.2 Soft tissue sarcomas	0.1	389		0.1	160	
4.4 Fibromatous neoplasms	0.6	1784		0.03	82	
4.5 Liposarcoma	0.3	856		0.04	104	
4.6 Synovial sarcoma	0.2	648		0.1	228	
4.7 Leiomyosarcoma	0.2	591		0.01	142	
4.8 Rhabdomyosarcoma	0.2	583		0.1	317	
4.9 Gastrointestinal stromal tumor	0.2	590		0.01	38	
4.10 Other soft tissue sarcoma	0.3	1092		0.1	386	
4.11 Other bone tumors	0.1	280		0.02	48	
**5. Blood and lymphatic vessel tumors**	0.6	1777	0.8	0.2	582	1.8
5.1 Specified (non- Kaposi sarcoma)	0.1	462		0.05	148	
5.2 Kaposi sarcoma	0.4	1315		0.1	434	
**6. Nerve sheath tumors(malignant)**	0.2	489	0.2	0.1	235	0.8
**7. Gonadal and related tumors**	**7.5**	24615	10.5	0.7	2006	6.4
7.1 Testis	**5.7**	18877		0.4	1049	
7.2 Ovary	**1.4**	4472		0.2	657	
7.3 Germ cell and trophoblastic (GCT)-CNS	0.1	369		0.02	72	
7.4 GCT (excluding CNS, testis, ovary)	0.3	889		0.1	226	
**8. Melanoma and Skin Carcinomas**	**5.3**	16935	7.2	0.3	810	2.6
**9. Carcinomas**	**41.1**	128599	54.8	**5.5**	14672	47.1
9.1 Thyroid carcinoma	**11.1**	35664		0.2	414	
9.2 Other carcinoma of head and neck	**1.5**	4684		0.3	694	
9.2.1 Nasopharyngeal carcinoma	0.2	610		0.04	109	
9.2.2 Lip, oral cavity and pharynx	0.7	2319		0.1	389	
9.2.3 Salivary gland	0.4	1191		0.03	83	
9.2.4 Other carcinoma of head and neck	0.2	564		0.04	113	
9.3 Carcinoma of gastrointestinal tract	**7.7**	24034		**2.2**	5946	
9.3.1 Carcinoma of esophagus	0.2	454		0.1	223	
9.3.2 Carcinoma of stomach	0.8	2426		0.4	1168	
9.3.3 Carcinoma of small intestine	0.3	833		0.05	133	
9.3.4 Carcinoma of colon	**3.4**	10649		0.7	1780	
9.3.5 Carcinoma of rectum	**1.7**	5100		0.4	1025	
9.3.6 Carcinoma of anus	0.2	575		0.05	129	
9.3.7 Carcinoma of liver and IBD	0.5	1421		0.3	681	
9.3.8 Carcinoma of gallbladder and other extrahepatic biliary	0.1	420		0.06	167	
9.3.9 Carcinoma of Pancreas	0.6	1999		0.2	550	

9.4 Carcinoma of lung, bronchus, and trachea	0.9	2658		0.3	886	
9.5 Carcinoma of skin	0.1	267		0.01	18	
9.6 Carcinoma of breast	**11.3**	34496		**1.3**	3437	
9.7 Carcinoma of genital sites (non ovary or testis)	**5.2**	16220		0.7	1926	
9.7.1 Carcinoma of uterine cervix	**3.0**	9421		0.5	1450	
9.8 Carcinoma of urinary tract	**2.9**	9090		0.3	760	
9.8.1 Carcinoma of kidney	**2.4**	7284		0.2	580	
9.8.2 Carcinoma of bladder	0.6	1712		0.06	151	
9.9 Carcinoma of other and ill-defined sites, NOS	0.5	1486		0.2	591	
**10. Miscellaneous specified neoplasms**	0.3	907	0.4	0.1	273	0.9
**11. Unspecified Malignant Neoplasms, except CNS**	0.6	1905	0.8	0.2	590	1.9
Any cancer type, Total	73.5	234565	100	11.3	31181	100

All incidence and mortality rates were age-adjusted and reported per 100,000 persons.

Table S4. AYA cancer incidence and mortality by sex, 2016-2021

	Incidence						Mortality					
**Cancer Subtype**	**Male (n=90,570)**			**Female (n=143,995)**			**Male (n=15,380)**			**Female (n=15,801)**		
	Rate	Count	%	Rate	Count	%	Rate	Count	%	Rate	Count	%
**1.Leukemias**	5.1	8405	9.3	4.3	6761	4.7	1.5	2155	14.0	1.0	1384	8.8
1.1 Acute lymphoid leukemia	1.5	2434		0.8	1279		0.6	877		0.3	462	
1.2 Acute myeloid leukemia	1.2	1999		1.3	2015		0.5	750		0.5	633	
1.3 Chronic myeloid leukemia	0.9	1442		0.6	959		0.1	181		0.05	75	
1.4 Other and unspecified leukemia	0.5	752		0.4	554		0.1	137		0.06	81	
**2.Lymphomas**	8.4	13820	15.3	7.1	11310	7.9	1.2	1709	11.1	0.7	900	5.7
2.1 non-Hodgkin lymphoma	4.2	6868		3.2	5010		0.8	1068		0.4	536	
2.2 Hodgkin lymphoma	3.4	5727		3.4	5457		0.3	429		0.2	248	
**3.CNS and Other Intracranial and Intraspinal Neoplasms**	3.1	5037	5.6	2.4	3756	2.6	1.3	1857	12.1	0.9	1190	7.5
3.1 Astrocytoma (Astroglia and related neoplasms)	2.8	4553		2.2	3414		1.1	1626		0.8	1045	
3.1.1 Oligodendrogliomas	0.4	697		0.3	533		0.1	200		0.1	156	
3.1.2 Glioblastoma (invasive)	0.7	1087		0.4	645		0.4	617		0.2	330	
3.1.3 Ependymoma (invasive)	0.2	285		0.2	282		0.04	59		0.03	49	
3.1.4 Other astrocytoma/astroglial neoplasms	1.5	2484		1.2	1954		0.5	750		0.4	510	
3.2 Medulloblastoma	0.2	268		0.1	149		0.1	142		0.05	76	
**4. Sarcomas**	3.3	5360	5.9	3.1	4889	3.3	1.1	1634	10.6	0.9	1184	7.5
4.1 Osteosarcoma	0.4	732		0.3	468		0.3	368		0.2	235	
4.2 Chondrosarcoma	0.2	322		0.2	294		0.03	45		0.02	30	
4.3 Ewing tumor	0.4	595		0.2	373		0.2	278		0.1	175	
4.3.1 bone tumors	0.2	383		0.1	196		0.1	185		0.1	108	
4.3.2 Soft tissue sarcomas	0.1	212		0.1	177		0.1	93		0.05	67	
4.4 Fibromatous neoplasms	0.5	878		0.6	906		0.03	44		0.03	38	
4.5 Liposarcoma	0.3	500		0.2	356		0.05	67		0.03	37	
4.6 Synovial sarcoma	0.2	310		0.2	338		0.1	134		0.1	94	
4.7 Leiomyosarcoma	0.1	182		0.3	409		0.03	38		0.1	104	
4.8 Rhabdomyosarcoma	0.2	331		0.2	252		0.1	202		0.1	115	
4.9 Gastrointestinal stromal tumor	0.2	295		0.2	295		0.02	24		0.01	14	
4.10 Other soft tissue sarcoma	0.3	496		0.4	596		0.1	186		0.1	200	

4.11 Other bone tumors	0.1	147		0.1	133		0.02	28		0.01	20	
**5. Blood and lymphatic vessel tumors**	0.9	1505	1.7	0.2	272	0.2	0.4	508	3.3	0.05	74	0.5
5.1.1 Specified (non-Kaposi sarcoma)	0.1	227		0.2	235		0.1	83		0.05	65	
5.1.2 Kaposi sarcoma	0.8	1278		0.02	37		0.3	425		0.01	9	
**6. Nerve sheath tumors (malignant)**	0.2	264	0.3	0.1	225	0.2	0.1	135	0.9	0.1	100	0.6
**7. Gonadal and related tumors**	11.6	19693	21.7	3.1	4922	3.4	0.9	1289	8.4	0.5	717	4.5
7.1 Testis	11.1	18877		-	-		0.7	1049		-	-	
7.2 Ovary	-	-		2.9	4472		-	-		0.5	657	
7.3 Germ cell and trophoblastic (GCT)-CNS	0.2	322		0.03	47		0.01	58		0.01	14	
7.4 GCT (excluding CNS, testis, ovary)	0.3	494		0.3	395		0.1	182		0.03	44	
**8. Melanoma and Skin Carcinomas**	3.7	5939	6.6	7.0	10996	7.6	0.3	447	2.9	0.3	363	2.3
**9. Carcinomas**	18.6	29558	32.6	64.4	99041	68.8	3.8	5261	34.2	7.1	9411	59.6
9.1 Thyroid carcinoma	4.2	6861		18.2	28803		0.1	136		0.2	278	
9.2 Other carcinoma of head and neck	1.6	2596		1.3	2088		0.3	473		0.2	221	
9.2.1 Nasopharyngeal carcinoma	0.2	387		0.1	223		0.1	78		0.02	31	
9.2.2 Lip, oral cavity and pharynx	0.9	1366		0.6	953		0.2	257		0.1	132	
9.2.3 Salivary gland	0.3	491		0.4	700		0.04	58		0.02	25	
9.2.4 Other carcinoma of head and neck	0.2	352		0.1	212		0.06	80		0.02	33	
9.3 Carcinoma of gastrointestinal tract	7.6	12072		7.8	11962		2.5	3373		1.9	2573	
9.3.1 Carcinoma of esophagus	0.2	357		0.1	97		0.1	175		0.04	48	
9.3.2 Carcinoma of stomach	0.8	1192		0.8	1234		0.5	624		0.4	544	
9.3.3 Carcinoma of small intestine	0.3	398		0.3	435		0.05	71		0.05	62	
9.3.4 Carcinoma of colon	3.2	5054		3.6	5595		0.7	936		0.6	844	
9.3.5 Carcinoma of rectum	1.8	2744		1.6	2356		0.4	599		0.3	426	
9.3.6 Carcinoma of anus	0.2	353		0.2	222		0.07	91		0.03	38	
9.3.7 Carcinoma of liver and intrahepatic bile duct	0.5	846		0.4	575		0.3	439		0.2	242	
9.3.8 Carcinoma of gallbladder and other extrahepatic biliary	0.1	214		0.1	206		0.1	85		0.1	82	
9.3.9 Carcinoma of Pancreas	0.5	851		0.7	1148		0.2	313		0.2	237	
9.4 Carcinoma of lung, bronchus, and trachea	0.8	1237		0.9	1421		0.3	484		0.3	402	
9.5 Carcinoma of skin	0.1	136		0.1	131		0.06	12		0.001	6	
9.6 Carcinoma of breast	0.1	135		22.9	34361		0.01	13		2.6	3424	
9.7 Carcinoma of genital sites (non ovary or testis)	0.2	330		10.4	15890		0.03	35		1.4	1891	
9.7.1 Carcinoma of uterine cervix	-	-		6.1	9421		-	-		1.1	1450	
9.8 Carcinoma of urinary tract	3.5	5506		2.4	3584		0.3	462		0.2	298	
9.8.1 Carcinoma of kidney	2.7	4272		2.0	3012		0.3	371		0.2	209	
9.8.2 Carcinoma of bladder	0.7	1176		0.4	536		0.06	75		0.06	76	

9.9 Carcinoma of other and ill-defined sites	0.4	685		0.5	801		0.2	273		0.2	318	
**10. Miscellaneous specified neoplasms**	0.2	315	0.3	0.4	592	0.4	0.1	136	0.9	0.1	137	0.9
**11. Unspecified malignant neoplasms, except CNS**	0.4	674	0.7	0.8	1231	0.9	0.2	249	1.6	0.3	341	2.1
Any cancer type, Total	55.4	90570	100	92.8	143995	100	10.8	15380	100	11.8	15801	100
**Race/ethnicity**												
Non-Hispanic White	64.2	49847	55.0	104.4	76783	53.3	10.3	6686	43.5	11.1	6780	42.9
Non-Hispanic Black	**44.0***	8783	9.7	**80.0***	16119	11.2	**14.4***	2482	16.1	**16.1***	2792	17.7
Non-Hispanic American Indian/Asian pacific	**42.1***	7340	8.1	**79.1***	14442	10.0	**8.8***	1389	9.0	**9.2***	1511	9.6
Hispanic (All Races)	**50.6***	24600	27.2	**85.2***	36651	25.5	**10.9***	4823	31.4	**12.0***	4718	29.8
**Metropolitan status**												
Metropolitan	55.2	82596	91.2	92.1	131471	91.3	10.7	13845	90.0	11.6	14250	90.2
Non-Metropolitan	**56.7***	7883	8.7	**101.0***	12377	8.6	**12.3***	1504	9.8	**14.1***	1522	9.6
Unknown/missing (Alaska or Hawaii)	61.5	91	0.1	**117.9***	147	0.1	**21.7***	31	0.2	**23.1***	29	0.2

All incidence and mortality rates were age-adjusted and reported per 100,000 persons.
SEER*Stat reports AYAs with missing/unknown metropolitan/nonmetropolitan status from Alaska or Hawaii.

Table S5. AYA cancer demographics by age group, 2016-2021

	**Incidence**								
	**All**			**15-19 y**			**20-24 y**		
**Characteristics**	**Rate**	**Count**	**%**	**Rate**	**Count**	**%**	**Rate**	**Count**	**%**
**Sex**									
Male	55.4	90570	37.2	24.1	7865	52.4	36.0	12016	50.3
Female	**92.8***	143995	62.8	**22.9***	7151	47.6	**37.6***	11882	49.7
**Race/ethnicity**									
Non-Hispanic White	83.9	126630	54.0	25.7	7436	49.5	42.4	12645	53.0
Non-Hispanic Black	**62.4***	24902	10.6	**17.0***	1437	9.5	**23.9***	2126	8.9
Non-Hispanic American Indian/Asian Pacific	**61.2***	21782	9.3	**20.8***	1186	7.9	**31.9***	1960	8.2
Hispanic (All Races)	**67.1***	61251	26.1	**23.7***	4957	33.1	**35.6***	7167	29.9
**Metropolitan status**									
Metropolitan	73.5	214067	91.3	23.6	13658	90.9	36.9	21830	91.3
Non-Metropolitan	**77.8***	20260	8.6	22.4	1343	9.0	**35.0***	2044	8.6
Unknown/missing (Alaska or Hawaii)	**88.7***	238	0.1	23.0	15	0.1	39.0	24	0.1
**Total**	73.8	234565	100	23.5	15016	100	36.8	23898	100
	**25-29 y**			**30-34 y**			**35-39 y**		
	**Rate**	**Count**	**%**	**Rate**	**Count**	**%**	**Rate**	**Count**	**%**
**Sex**									
Male	49.7	17249	42.6	67.6	22962	36.2	93.2	30478	33.2
Female	**69.8***	23239	57.4	**123.5***	40523	63.8	**192.3***	61200	66.8
**Race/ethnicity**									
Non-Hispanic White	68.9	21817	53.9	109.0	34857	54.9	160.1	49875	54.4
Non-Hispanic Black	**43.8***	4102	10.1	**79.4***	6706	10.6	**134.6***	10531	11.5
Non-Hispanic American Indian/Asian Pacific	**47.1***	3531	8.7	**76.3***	6041	9.5	**119.4***	9064	9.9
Hispanic (All Races)	**56.7***	11038	27.3	**86.0***	15881	25.0	**123.6***	22208	24.2
**Metropolitan status**									

Metropolitan	59.4	37126	91.7	94.4	58086	91.5	140.9	83367	90.9
Non-Metropolitan	61.1	3318	8.2	**101.6***	5325	8.4	**154.7***	8230	9.0
Unknown/missing (Alaska or Hawaii)	70.3	44	0.1	**129.3***	74	0.1	167.5	81	0.1
Total	59.5	40488	100	95.0	63485	100	142.0	91678	100
	**Mortality**								
	**All**			**15-19 y**			**20-24 y**		
	**Rate**	**Count**	**%**	**Rate**	**Count**	**%**	**Rate**	**Count**	**%**
**Sex**									
Male	10.8	15380	49.3	4.3	1241	61.3	5.9	1713	61.6
Female	**11.8***	15801	50.7	**2.9***	781	38.7	**3.9***	1069	38.4
**Race/ethnicity**									
Non-Hispanic White	10.7	13466	43.2	3.2	770	38.1	4.1	1016	36.5
Non-Hispanic Black	**15.3***	5274	16.9	**4.1***	301	14.9	**5.3***	412	14.8
Non-Hispanic American Indian/Asian pacific	**9.0***	2900	9.3	3.6	186	9.2	4.0	219	7.9
Hispanic (All Races)	**11.4***	9541	30.6	**4.0***	765	37.8	**6.1***	1135	40.8
**Metropolitan status**									
Metropolitan	11.1	28095	90.1	3.6	1833	90.6	5.0	2557	91.9
Non-Metropolitan	**13.2***	3026	9.7	3.4	183	9.1	**4.2***	222	8.0
Unknown/missing (Alaska or Hawaii)	**22.4***	60	0.2	9.2	6	0.3	4.9	3	0.1
**Total**	11.3	31181	100	3.6	2022	100	4.9	2782	100
	**25-29 y**			**30-34 y**			**35-39 y**		
	**Rate**	**Count**	**%**	**Rate**	**Count**	**%**	**Rate**	**Count**	**%**
**Sex**									
Male	8.5	2602	56.0	12.7	3801	47.2	21.0	6023	44.0
Female	**7.0***	2041	44.0	**14.8***	4252	52.8	**27.4***	7658	56.0
**Race/ethnicity**									
Non-Hispanic White	7.1	1892	40.7	13.3	3600	44.7	23.5	6188	45.2
Non-Hispanic Black	**8.9***	731	15.7	**18.7***	1389	17.2	**35.5***	2441	17.8
Non-Hispanic American Indian/Asian pacific	6.7	453	9.8	**10.3***	737	9.2	**18.9***	1305	9.5
Hispanic (All Races)	**8.7***	1567	33.8	13.7	2327	28.9	22.6	3747	27.5
**Metropolitan status**									
Metropolitan	7.7	4194	90.3	13.5	7264	90.2	23.6	12247	89.5
Non-Metropolitan	**8.9***	435	9.4	**16.5***	774	9.6	**29.7***	1412	10.3
Unknown/missing (Alaska or Hawaii)	**22.4***	14	0.3	**26.2***	15	0.2	**45.5***	22	0.2
**Total**	7.8	4643	100	13.7	8053	100	24.2	13681	100

*: Significant at 0.05 level

All incidence and mortality rates were age-adjusted and reported per 100,000 persons.

SEER*Stat reports AYAs with missing/unknown metropolitan/nonmetropolitan status from Alaska or Hawaii.

Table S6. Trends in cancer incidence and mortality, 2016-2021.

**Cancer subtype**	**Incidence**		**Mortality**	
	**APC (95% CI)**	**P value**	**APC (95% CI)**	**P value**
**1.Leukemias**	0.2 (-2.5, 3.1)	0.82	**-2.0 (-3.0, -1.0)**	**0.006**
1.1 Acute lymphoid leukemia	2.1 (-0.3, 4.7)	0.07	-1.4 (-4.3, 1.7)	0.27
1.2 Acute myeloid leukemia	0.5 (-4.4, 5.7)	0.79	-3.3 (-6.8, 0.4)	0.066
1.3 Chronic myeloid leukemia	-1.2 (-3.4, 1.0)	0.21	-2.5 (-10.1, 5.7)	0.43
1.4 Other and unspecified leukemia	1.3 (-4.8, 7.8)	0.59	5.2 (-4.6, 16.1)	0.222

Trends in Cancer Incidence and Mortality in US Adolescents and Young Adults, 2016-2021, Li Zhang, Online Supplement Document

**2.Lymphomas**	**-1.4 (-2.7, -0.1)**	**0.04**	-1.6 (-5.1, 2.0)	0.28
2.1 non-Hodgkin lymphoma	**-2.3 (-3.3, -1.3)**	**0.003**	-3.5 (-7.8, 1.0)	0.10
2.2 Hodgkin lymphoma	-0.2 (-2.5, 2.2)	0.82	4.8 (-1.9, 12.1)	0.121
**3.CNS and Other Intracranial and Intraspinal Neoplasms (All behavior)**	-1.0 (-2.5, 0.4)	0.12	0.5 (-4.4, 5.6)	0.805
3.1 Astrocytoma (Astroglia and related neoplasms)	-0.8 (-2.6, 1.1)	0.29	0.7 (-4.0, 5.6)	0.713
3.1.1 Oligodendrogliomas	0.3 (-4.3, 5.0)	0.88	-0.2 (-4.5, 4.3)	0.91
3.1.2 Glioblastoma (invasive)	-1.8 (-9.8, 7.0)	0.60	2.2 (-2.2, 6.7)	0.243
3.1.3 Ependymoma (invasive)	**-5.4 (-10.4, -0.2)**	**0.045**	1.6 (-10.5, 15.5)	0.742
3.1.4 Other astrocytoma/astroglial neoplasms	-0.001 (-3.0, 3.1)	0.99	-0.3 (-6.2, 6.1)	0.911
3.2 Medulloblastoma	-2.6 (-7.0, 2.1)	0.20	1.8 (-8.3, 13.0)	0.66
**4. Sarcomas**	-2.0 (-4.3, 0.4)	0.08	0.2 (-1.8, 2.3)	0.78
4.1 Osteosarcoma	-1.8 (-5.7, 2.4)	0.30	-0.4 (-7.6, 7.3)	0.88
4.2 Chondrosarcoma	-1.1 (-8.7, 7.1)	0.71	0.8 (-17.2, 22.7)	0.918
4.3 Ewing tumor	-1.2 (-5.5, 3.3)	0.49	-0.6 (-9.3, 9.0)	0.87
4.3.1 bone tumors	-0.8 (-3.8, 2.3)	0.52	-2.5 (-8.6, 4.1)	0.342
4.3.2 Soft tissue sarcomas	-1.9 (-12.4, 9.9)	0.67	3.2 (-12.7, 21.9)	0.632
4.4 Fibromatous neoplasms	-7.9 (-16.7, 1.8)	0.09	-8.4 (-20.6, 5.8)	0.17
4.5 Liposarcoma	**-3.6 (-6.5, -0.7)**	**0.03**	1.8 (-18.3, 26.7)	0.834
4.6 Synovial sarcoma	-2.5 (-6.7, 2.0)	0.19	0.3 (-9.1, 10.6)	0.94
4.7 Leiomyosarcoma	-5.0 (-11.0, 1.5)	0.10	-8.7 (-18.5, 2.2)	0.09
4.8 Rhabdomyosarcoma	2.0 (-4.0, 8.5)	0.42	5.2 (-5.5, 17.0)	0.26
4.9 Gastrointestinal stromal tumor	10.4 (-6.3, 30.0)	0.17	1.9 (-21.0, 31.5)	0.84
4.10 Other soft tissue sarcoma	-1.0 (-6.3, 4.6)	0.64	3.5 (-2.2, 9.5)	0.17
4.11 Other bone tumors	-0.2 (-8.0, 8.3)	0.95	11.1 (-3.8, 28.3)	0.11
**5. Blood and lymphatic vessel tumors**	0.2 (-6.4, 7.3)	0.93	-2.0 (-5.8, 2.0)	0.24
5.1.1 Specified (non- Kaposi sarcoma)	**8.0 (2.2, 14.2)**	**0.02**	9.3 (-9.6, 32.2)	0.26
5.1.2 Kaposi sarcoma	-2.4 (-9.6, 5.4)	0.40	-5.1 (-11.4, 1.7)	0.11
**6. Nerve sheath tumors(malignant)**	2.3 (-2.0, 6.8)	0.21	**13.2 (0.2, 27.8)**	**0.047**
**7. Gonadal and related tumors**	-0.4 (-1.2, 0.4)	0.26	2.3 (-0.4, 5.1)	0.08
7.1 Testis	-0.7 (-1.8, 0.5)	0.20	4.8 (-0.9, 10.9)	0.08
7.2 Ovary	1.4 (-0.2, 3.1)	0.08	-0.6 (-6.8, 5.9)	0.80
7.3 Germ cell and trophoblastic (GCT)-CNS	1.0 (-4.3, 6.7)	0.63	6.1 (-11.3, 26.8)	0.41
7.4 GCT (excluding CNS, testis, ovary)	-4.5 (-10.5, 1.9)	0.12	-1.1 (-7.2, 5.5)	0.66
**8. Melanoma**	**-4.6 (-8.2, -1.0)**	**0.025**	**-7.7 (-14.4, -0.4)**	**0.043**
**9. Carcinomas**	-0.4 (-2.7, 1.9)	0.64	-0.5 (-1.8, 0.8)	0.344
9.1 Thyroid carcinoma	-2.1 (-5.9, 1.8)	0.20	2.7 (-2.1, 7.8)	0.193
9.2 Other carcinoma of head and neck	-2.0 (-4.8, 0.9)	0.13	1.1 (-5.4, 8.0)	0.67
9.2.1 Nasopharyngeal carcinoma	0.03 (-5.4, 5.8)	0.99	0.4 (-17.3, 21.9)	0.95
9.2.2 Lip, oral cavity and pharynx	-2.6 (-5.6, 0.5)	0.078	1.4 (-8.7, 12.6)	0.73
9.2.3 Salivary gland	-1.6 (-4.5, 1.5)	0.221	5.0 (-6.6, 18.0)	0.311
9.2.4 Other carcinoma of head and neck	-2.3 (-11.3, 7.5)	0.53	-1.5 (-12.6, 11.0)	0.75
9.3 Carcinoma of gastrointestinal tract	**1.3 (0.3, 2.2)**	**0.019**	0.04 (-0.7, 0.8)	0.89
9.3.1 Carcinoma of esophagus	2.9 (-7.1, 13.9)	0.49	5.8 (-0.9, 13.1)	0.08
9.3.2 Carcinoma of stomach	-1.7 (-4.4, 1.2)	0.177	-0.6 (-4.7, 3.5)	0.69
9.3.3 Carcinoma of small intestine	2.1 (-3.9, 8.4)	0.39	-4.2 (-14.0, 6.8)	0.34
9.3.4 Carcinoma of colon	1.1 (-0.6, 2.8)	0.15	1.3 (-3.8, 6.5)	0.53
9.3.5 Carcinoma of rectum	**2.8 (0.6, 5.1)**	**0.024**	-0.2 (-3.1, 2.8)	0.88
9.3.6 Carcinoma of anus	3.3 (-6.3, 13.9)	0.41	2.6 (-13.9, 22.3)	0.71
9.3.7 Carcinoma of liver and IBD	-1.1 (-6.4, 4.5)	0.598	**-4.1 (-6.9, -1.2)**	**0.018**
9.3.8 Carcinoma of gallbladder and other extrahepatic biliary	0.9 (-1.8, 3.7)	0.41	-0.6 (-16.5, 18.4)	0.93
9.3.9 Carcinoma of Pancreas	2.8 (-3.7, 9.9)	0.30	1.8 (-5.6, 9.8)	0.55
9.4 Carcinoma of lung, bronchus, and trachea	-3.9 (-7.7, 0.1)	0.052	-2.3 (-6.9, 2.6)	0.262

9.5 Carcinoma of skin	-4.2 (-11.7, 3.9)	0.216	19.3 (-15.2, 68.0)	0.225
9.6 Carcinoma of breast	1.0 (-0.8, 2.9)	0.20	-1.6 (-5.1, 2.1)	0.291
9.7 Carcinoma of genital sites (non ovary or testis)	-1.4 (-4.5, 1.9)	0.31	-0.4 (-5.9, 5.5)	0.87
9.7.1 Carcinoma of uterine cervix	-3.1 (-6.4, 0.3)	0.07	-2.6 (-8.0, 3.1)	0.267
9.8 Carcinoma of urinary tract	-1.0 (-4.8, 3.0)	0.51	-3.6 (-8.6, 1.7)	0.13
9.8.1 Carcinoma of kidney	-0.4 (-4.3, 3.7)	0.80	**-5.5 (-9.8, -1.1)**	**0.026**
9.8.2 Carcinoma of bladder	-3.2 (-9.0, 3.0)	0.22	2.3 (-9.7, 16.0)	0.64
9.9 Carcinoma of other and ill-defined sites, NOS	**4.8 (0.6, 9.2)**	**0.03**	1.9 (-2.0, 6.0)	0.25
**10. Miscellaneous specified neoplasms**	6.9 (-6.3, 22.1)	0.23	4.1 (-0.3, 8.8)	0.061
**11. Unspecified Malignant Neoplasms, except CNS**	**-4.0 (-6.4, -1.4)**	**0.01**	**3.1 (0.4, 5.9)**	**0.034**
Any cancer type, Total	-0.9 (-2.8, 1.1)	0.29	-0.45 (-0.9, 0.02)	0.06

APC: annual percentage change

Table S7. Trends in cancer incidence and mortality for males and females, 2016-2021

**Cancer Subtype**	**Incidence**				**Mortality**			
	**Male**		**Female**		**Male**		**Female**	
	**APC (95% CI)**	**P value**	**APC (95% CI)**	**P value**	**APC (95% CI)**	**P value**	**APC (95% CI)**	**P value**
**1.Leukemias**	0.01 (-3.5, 3.7)	0.99	0.5 (-1.8, 2.9)	0.57	**-1.5 (-2.8, -0.1)**	**0.04**	**-2.8 (-4.8, -0.6)**	**0.02**
1.1 Acute lymphoid leukemia	1.3 (-2.5, 5.3)	0.39	**3.7 (2.0, 5.4)**	**0.004**	-2.1 (-5.8, 1.8)	0.21	-0.1 (-3.5, 3.4)	0.92
1.2 Acute myeloid leukemia	0.6 (-6.6, 8.3)	0.85	0.5 (-5.5, 6.8)	0.85	-2.4 (-5.3, 0.7)	0.09	-4.3 (-10.1, 2.0)	0.13
1.3 Chronic myeloid leukemia	-2.1 (-6.4, 2.3)	0.25	0.2 (-3.9, 4.4)	0.92	-1.7 (-13.2, 11.4)	0.72	-5.2 (-23.0, 16.7)	0.51
1.4 Other and unspecified leukemia	2.1 (-4.0, 8.5)	0.40	0.03 (-8.9, 9.9)	0.99	**8.9 (3.5, 14.6)**	**0.01**	1.2 (-21.7, 30.9)	0.90
**2.Lymphomas**	-1.4 (-3.5, 0.7)	0.13	**-1.4 (-2.6, -0.1)**	**0.04**	-3.3 (-6.7, 0.3)	0.07	1.5 (-4.0, 7.2)	0.50
2.1 non-Hodgkin lymphoma	**-2.4 (-3.6, -1.2)**	**0.005**	-2.2 (-4.6, 0.3)	0.07	**-5.7 (-10.1, -1.1)**	**0.03**	0.8 (-3.8, 5.7)	0.65
2.2 Hodgkin lymphoma	-0.2 (-3.7, 3.4)	0.89	-0.2 (-2.6, 2.2)	0.79	4.7 (-2.3, 12.2)	0.14	4.4 (-9.5, 20.4)	0.45
**3.CNS and Other Intracranial and Intraspinal Neoplasms (All behavior)**	-1.1 (-2.4, 0.4)	0.11	-1.0 (-3.0, 1.0)	0.23	1.9 (-2.5, 6.5)	0.30	-1.8 (-7.5, 4.4)	0.47
3.1 Astrocytoma (Astroglia and related neoplasms)	-0.9 (-2.4, 0.7)	0.20	-0.8 (-3.3, 1.8)	0.45	2.1 (-2.1, 6.6)	0.24	-1.5 (-7.6, 4.8)	0.52
3.1.1 Oligodendrogliomas	0.5 (-2.7, 3.8)	0.71	-0.05 (-8.0, 8.6)	0.99	-1.0 (-10.8, 9.8)	0.79	1.4 (-9.0, 13.0)	0.74
3.1.2 Glioblastoma (invasive)	-0.6 (-9.4, 9.2)	0.87	-3.9 (-13.6, 6.8)	0.35	**4.7 (1.1, 8.5)**	**0.02**	-2.3 (-12.8, 9.4)	0.60
3.1.3 Ependymoma (invasive)	-5.2 (-11.6, 1.7)	0.10	**-5.6 (-10.0, -1.0)**	**0.03**	8.5 (-9.1, 29.4)	0.27	-5.5 (-20.0, 11.6)	0.40
3.1.4 Other astrocytoma/astroglial neoplasms	-0.7 (-4.3, 3.1)	0.65	0.8 (-2.9, 4.7)	0.57	0.7 (-7.9, 10.0)	0.85	-1.6 (-5.5, 2.4)	0.33
3.2 Medulloblastoma	-3.1 (-9.6, 3.8)	0.27	-1.9 (-10.1, 7.0)	0.57	3.5 (-7.8, 16.1)	0.46	-1.3 (-11.6, 10.3)	0.76
**4. Sarcomas**	-1.4 (-4.1, 1.4)	0.24	-2.7 (-6.8, 1.6)	0.16	0.5 (-1.7, 2.7)	0.56	-0.2 (-5.7, 5.7)	0.94
4.1 Osteosarcoma	-1.2	0.26	-2.9	0.45	0.3	0.92	-1.5	0.68

	(-3.6, 1.3)		(-11.7, 6.9)		(-8.1, 9.5)		(-10.3, 8.2)	
4.2 Chondrosarcoma	2.3 (-9.9, 16.3)	0.64	-4.9 (-13.9, 4.9)	0.23	2.8 (-13.7, 22.4)	0.69	-0.1 (-26.0, 34.8)	0.99
4.3 Ewing tumor	-1.0 (-6.9, 5.3)	0.68	-1.4 (-13.3, 12.1)	0.77	-0.7 (-6.0, 4.8)	0.73	0.1 (-21.1, 26.9)	0.99
4.3.1 bone tumors	-1.1 (-8.8, 7.3)	0.73	-0.3 (-8.1, 8.1)	0.92	-2.9 (-9.2, 3.9)	0.29	-1.7 (-15.0, 13.6)	0.76
4.3.2 Soft tissue sarcomas	-1.2 (-10.6, 9.3)	0.76	-2.2 (-19.4, 18.8)	0.77	4.4 (-8.4, 19.0)	0.42	3.7 (-30.8, 55.3)	0.82
4.4 Fibromatous neoplasms	**-6.8 (-13.2, -0.01)**	**0.048**	-8.6 (-22.9, 8.3)	0.22	-8.8 (-33.5, 25.0)	0.46	-6.9 (-24.9, 15.3)	0.40
4.5 Liposarcoma	-2.8 (-10.5, 5.6)	0.40	-4.7 (-11.9, 3.0)	0.16	5.1 (-10.1, 22.9)	0.43	-2.7 (-31.8, 38.7)	0.84
4.6 Synovial sarcoma	-0.7 (-8.3, 7.7)	0.83	-4.1 (-8.6, 0.7)	0.08	-1.7 (-17.5, 17.1)	0.80	2.7 (-11.7, 19.4)	0.65
4.7 Leiomyosarcoma	-10.6 (-20.4, 0.5)	0.06	-2.1 (-11.2, 7.9)	0.57	-12.2 (-39.6, 27.6)	0.39	-6.2 (-16.8, 5.8)	0.22
4.8 Rhabdomyosarcoma	-0.7 (-9.8, 9.2)	0.84	6.3 (-2.7, 16.0)	0.13	3.8 (-11.0, 21.1)	0.54	8.0 (-6.5, 24.8)	0.21
4.9 Gastrointestinal stromal tumor	8.9 (-11.4, 33.9)	0.31	11.9 (-2.2, 28.0)	0.08	-6.6 (-25.6, 17.3)	0.45	14.0 (-25.6, 74.6)	0.44
4.10 Other soft tissue sarcoma	-1.0 (-5.3, 3.6)	0.59	-1.2 (-10.3, 9.0)	0.76	**4.3 (0.9, 7.9)**	**0.03**	2.8 (-6.9, 13.5)	0.49
4.11 Other bone tumors	1.6 (-9.0, 13.5)	0.71	-2.0 (-10.2, 7.0)	0.56	9.6 (-10.6, 34.4)	0.28	11.9 (-12.7, 43.4)	0.28
**5. Blood and lymphatic vessel tumors**	-1.2 (-8.7, 6.9)	0.69	**8.0 (4.0, 12.2)**	**0.005**	-4.5 (-10.0, 1.4)	0.10	19.8 (-12.4, 64.0)	0.17
5.1.1 Specified (non- Kaposi sarcoma)	8.2 (-1.4, 18.8)	0.08	7.5 (-2.0, 17.9)	0.10	0.9 (-9.5, 12.5)	0.83	21.5 (-7.9, 60.3)	0.12
5.1.2 Kaposi sarcoma	-2.8 (-10.5, 5.7)	0.40	2.8 (-34.6, 61.5)	0.88	-5.3 (-13.0, 3.0)	0.15	-	-
**6. Nerve sheath tumors(malignant)**	2.3 (-8.8, 14.8)	0.61	2.0 (-10.7, 16.6)	0.70	**16.8 (1.0, 35.1)**	**0.041**	7.7 (-17.7, 40.9)	0.49
**7. Gonadal and related tumors**	-0.8 (-1.8, 0.3)	0.11	0.9 (-0.7, 2.5)	0.19	3.7 (-0.9, 8.4)	0.1	-0.1 (-5.8, 6.0)	0.97
7.1 Testis	-0.7 (-1.9, 0.5)	0.16	-	-	4.8 (-1.0, 10.8)	0.08	-	-
7.2 Ovary	-	-	1.5 (-0.1, 3.2)	0.06	-	-	-0.5 (-6.6, 6.1)	0.85
7.3 Germ cell and trophoblastic (GCT)-CNS	0.9 (-4.9, 7.0)	0.71	-0.04 (-20.9, 26.3)	0.99	0.9 (-21.6, 30.0)	0.92	-	-
7.4 GCT (excluding CNS, testis, ovary)	-3.4 (-13.1, 7.4)	0.42	**-5.8 (-8.9, -2.6)**	**0.008**	-1.0 (-9.5, 8.4)	0.78	-1.5 (-13.3, 11.9)	0.76
**8. Melanoma and Skin Carcinomas**	**-6.1 (-9.9, -2.1)**	**0.014**	**-3.8 (-7.3, -0.1)**	**0.047**	**-7.6 (-13.1, -1.7)**	**0.02**	-7.8 (-18.2, 4.0)	0.14
**9. Carcinomas**	-0.2 (-2.1, 1.7)	0.80	-0.4 (-2.8, 2.1)	0.68	0.1 (-2.0, 2.3)	0.88	-0.8 (-2.1, 0.6)	0.19
9.1 Thyroid carcinoma	-1.5 (-4.1, 1.1)	0.18	-2.2 (-6.3, 2.2)	0.23	-3.9 (-12.7, 5.8)	0.32	**6.3 (0.1, 12.8)**	**0.048**
9.2 Other carcinoma of head and neck	-2.5 (-5.5, 0.6)	0.09	-1.4 (-5.5, 2.9)	0.41	1.5 (-4.8, 8.3)	0.55	-0.4 (-13.0, 14.2)	0.95
9.2.1 Nasopharyngeal carcinoma	-0.9 (-7.3, 6.0)	0.74	1.7 (-5.4, 9.2)	0.56	-7.0 (-23.0, 12.5)	0.35	-	-

9.2.2 Lip, oral cavity and pharynx	-3.0 (-6.8, 1.0)	0.11	-2.1 (-8.7, 5.0)	0.45	2.2 (-11.6, 18.0)	0.70	-0.8 (-10.4, 9.7)	0.83
9.2.3 Salivary gland	-2.2 (-6.9, 2.8)	0.28	-1.2 (-7.6, 5.7)	0.66	11.3 (-4.9, 30.2)	0.13	-1.7 (-37.1, 53.6)	0.92
9.2.4 Other carcinoma of head and neck	-3.0 (-15.1, 10.7)	0.56	-1.5 (-11.0, 9.0)	0.70	3.0 (-12.7, 21.4)	0.65	-12.0 (-31.1, 12.4)	0.22
9.3 Carcinoma of gastrointestinal tract	0.8 (-1.2, 2.8)	0.33	1.3 (-0.6, 3.5)	0.12	0.6 (-2.3, 3.6)	0.59	-0.7 (-4.5, 3.2)	0.63
9.3.1 Carcinoma of esophagus	1.5 (-10.7, 15.4)	0.76	6.9 (-5.3, 20.8)	0.20	7.8 (-4.6, 21.8)	0.16	-2.6 (-27.2, 30.4)	0.81
9.3.2 Carcinoma of stomach	-3.3 (-7.4, 1.0)	0.1	-0.1 (-2.7, 2.5)	0.90	-1.4 (-8.7, 6.5)	0.64	0.2 (-3.3, 3.9)	0.87
9.3.3 Carcinoma of small intestine	1.2 (-5.7, 8.7)	0.65	2.9 (-4.1, 10.5)	0.32	**-10.2 (-16.6, -3.2)**	**0.02**	2.4 (-16.0, 24.9)	0.76
9.3.4 Carcinoma of colon	1.0 (-1.3, 3.3)	0.30	1.2 (-2.5, 5.0)	0.43	2.0 (-2.4, 6.7)	0.28	0.4 (-7.2, 8.6)	0.89
9.3.5 Carcinoma of rectum	2.8 (-1.2, 6.9)	0.13	2.8 (-0.7, 6.5)	0.09	2.3 (-3.7, 8.8)	0.35	-3.3 (-10.1, 4.1)	0.27
9.3.6 Carcinoma of anus	5.7 (-2.3, 14.4)	0.12	-0.5 (-15.6, 17.2)	0.93	0.2 (-15.6, 18.8)	0.98	4.2 (-24.3, 43.4)	0.74
9.3.7 Carcinoma of liver and intrahepatic bile ducts	-1.7 (-8.9, 6.0)	0.56	-0.04 (-8.0, 8.6)	0.99	-2.8 (-9.6, 4.5)	0.34	-6.1 (-12.7, 1.0)	0.08
9.3.8 Carcinoma of gallbladder and other extrahepatic biliary	-1.6 (-11.1, 9.1)	0.69	3.2 (-6.8, 14.2)	0.44	7.0 (-14.6, 34.1)	0.45	-8.0 (-21.4, 7.7)	0.22
9.3.9 Carcinoma of Pancreas	4.4 (-3.9, 13.4)	0.22	1.7 (-4.9, 8.8)	0.52	-0.3 (-6.9, 6.8)	0.91	4.4 (-6.2, 16.2)	0.33
9.4 Carcinoma of lung, bronchus, and trachea	-2.5 (-5.9, 0.9)	0.11	**-5.1 (-9.8, -0.1)**	**0.048**	-3.4 (-11.3, 5.2)	0.33	-1.0 (-4.9, 3.1)	0.53
9.5 Carcinoma of skin	-5.0 (-20.5, 13.6)	0.47	-2.4 (-18.9, 17.5)	0.74	-	-	-	-
9.6 Carcinoma of breast	6.7 (-7.6, 23.2)	0.28	1.2 (-0.7, 3.1)	0.16	-2.7 (-33.1, 41.5)	0.85	-1.4 (-5.0, 2.4)	0.36
9.7 Carcinoma of genital sites (non ovary or testis)	4.5 (-10.6, 22.0)	0.48	-1.3 (-4.3, 1.7)	0.29	1.8 (-19.2, 28.4)	0.84	-0.2 (-5.7, 5.5)	0.91
9.7.1 Carcinoma of uterine cervix	**-**	-	-2.9 (-6.2, 0.5)	0.08	-	-	-2.4 (-7.8, 3.3)	0.29
9.8 Carcinoma of urinary tract	-0.6 (-4.8, 3.8)	0.73	-1.8 (-5.2, 1.8)	0.23	-2.6 (-8.0, 3.1)	0.27	-5.1 (-15.2, 6.1)	0.26
9.8.1 Carcinoma of kidney	-0.2 (-4.9, 4.7)	0.91	-0.7 (-3.9, 2.6)	0.56	**-5.1 (-9.1, -0.9)**	**0.03**	-6.3 (-17.4, 6.3)	0.23
9.8.2 Carcinoma of bladder	-2.0 (-7.7, 4.2)	0.42	-6.1 (-12.6, 0.8)	0.07	1.9 (-17.3, 25.5)	0.82	1.1 (-15.1, 20.2)	0.88
9.9 Carcinoma of other and ill-defined sites, NOS	4.0 (-1.6, 9.9)	0.12	**5.6 (0.2, 11.2)**	**0.04**	3.4 (-1.7, 8.7)	0.14	0.6 (-9.2, 11.5)	0.88
**10. Miscellaneous specified neoplasms**	4.7 (-9.6, 21.2)	0.43	8.1 (-5.5, 23.8)	0.18	8.8 (-6.4, 26.6)	0.20	0.6 (-10.7, 13.3)	0.90
**11. Unspecified Malignant Neoplasms, except CNS**	0.7 (-4.0, 5.5)	0.72	**-6.3 (-10.9, -1.5)**	**0.02**	8.3 (-3.3, 21.2)	0.12	-0.1 (-4.9, 4.9)	0.96
Any cancer type, Total	-1.0 (-2.7, 0.7)	0.18	-0.7 (-2.9, 1.5)	0.41	-0.01 (-0.7, 0.7)	0.975	**-0.8 (-1.2, -0.5)**	**0.003**

APC: annual percentage change

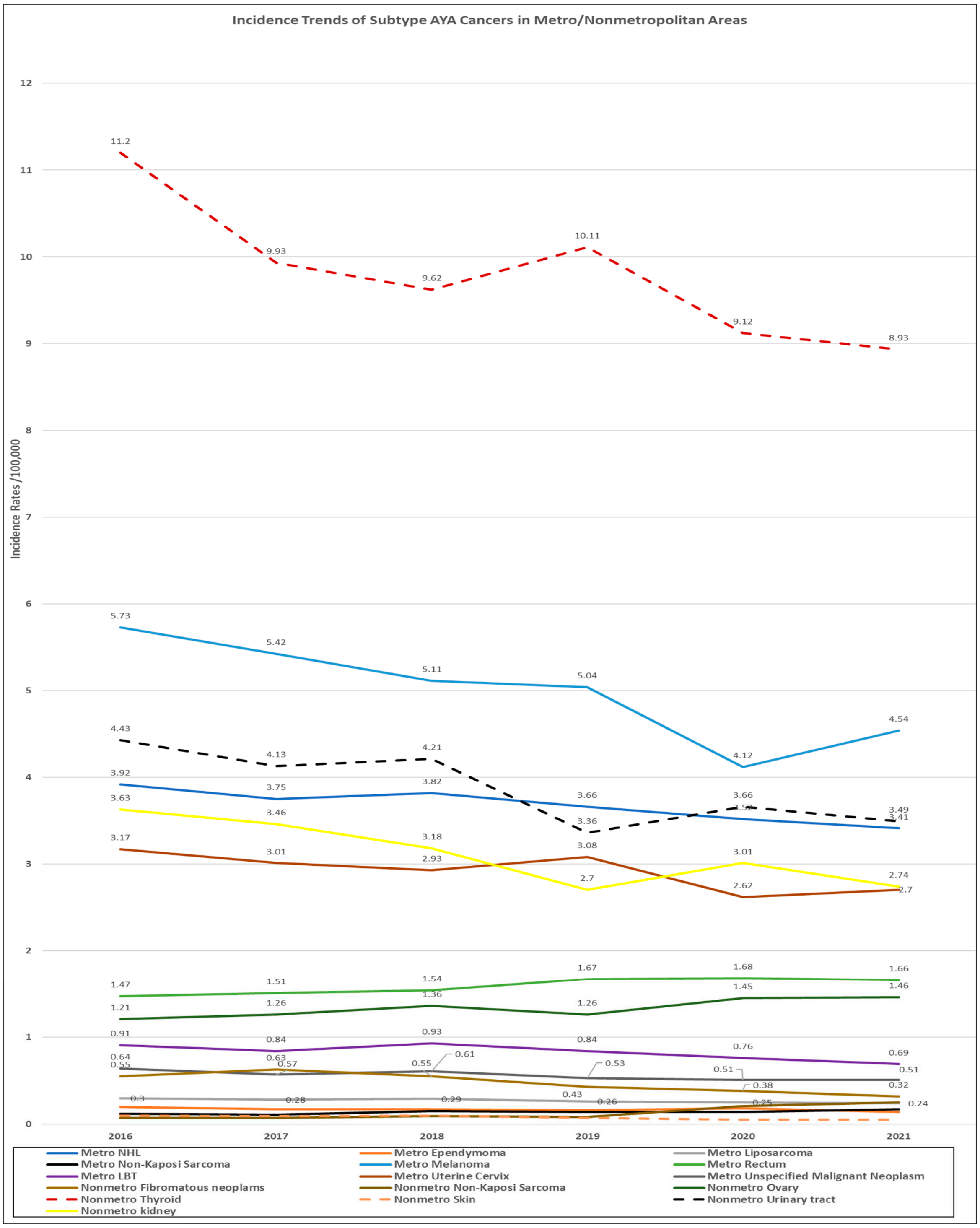

Figure S1. Incidence trends of subtype cancers by metro/nonmetropolitan status when considering first primary cancer diagnosis (significant change of trends)

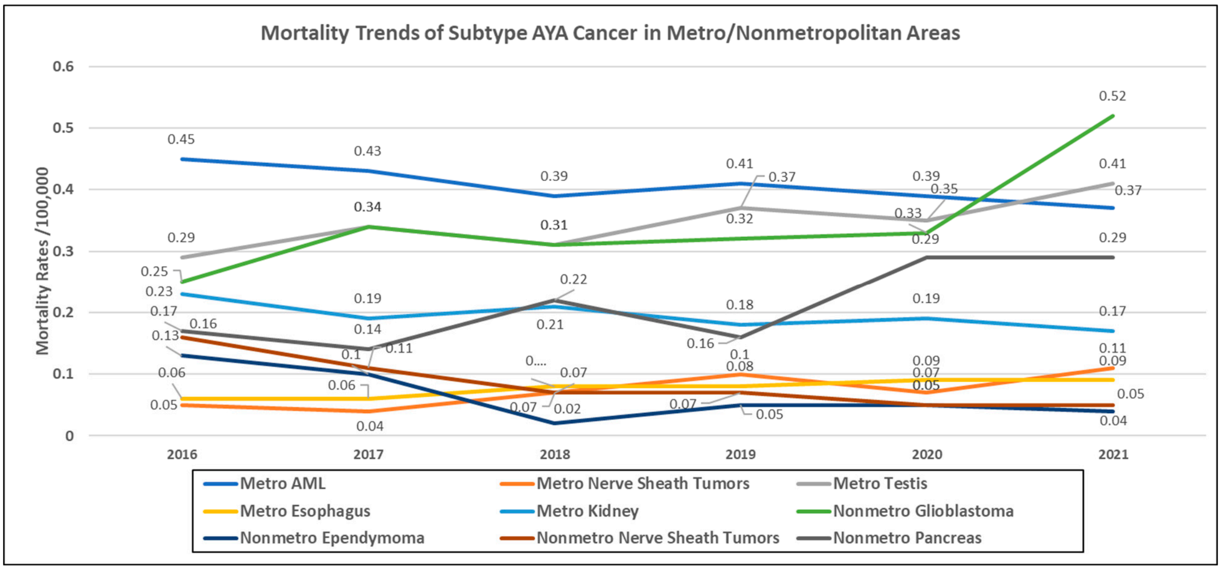

Figure S2. Mortality trends of subtype cancers by metro/nonmetropolitan status when considering first primary cancer death (significant change of trends)

Trends in Cancer Incidence and Mortality in US Adolescents and Young Adults, 2016-2021, Li Zhang, Online Supplement Document

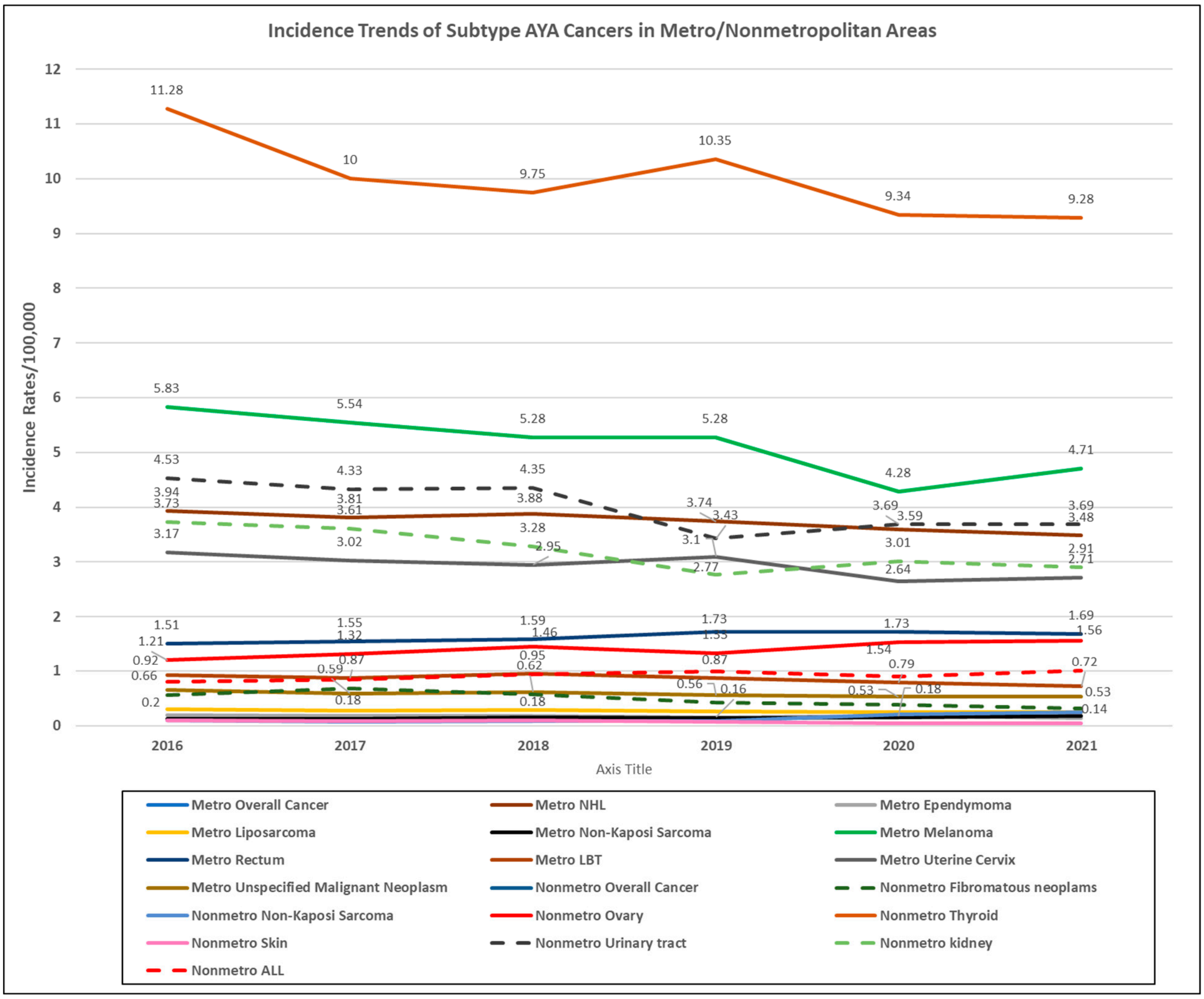

Figure S3. Incidence trends of subtype cancers by metro/nonmetropolitan status when considering multiple primary cancer diagnoses (significant change of trends)

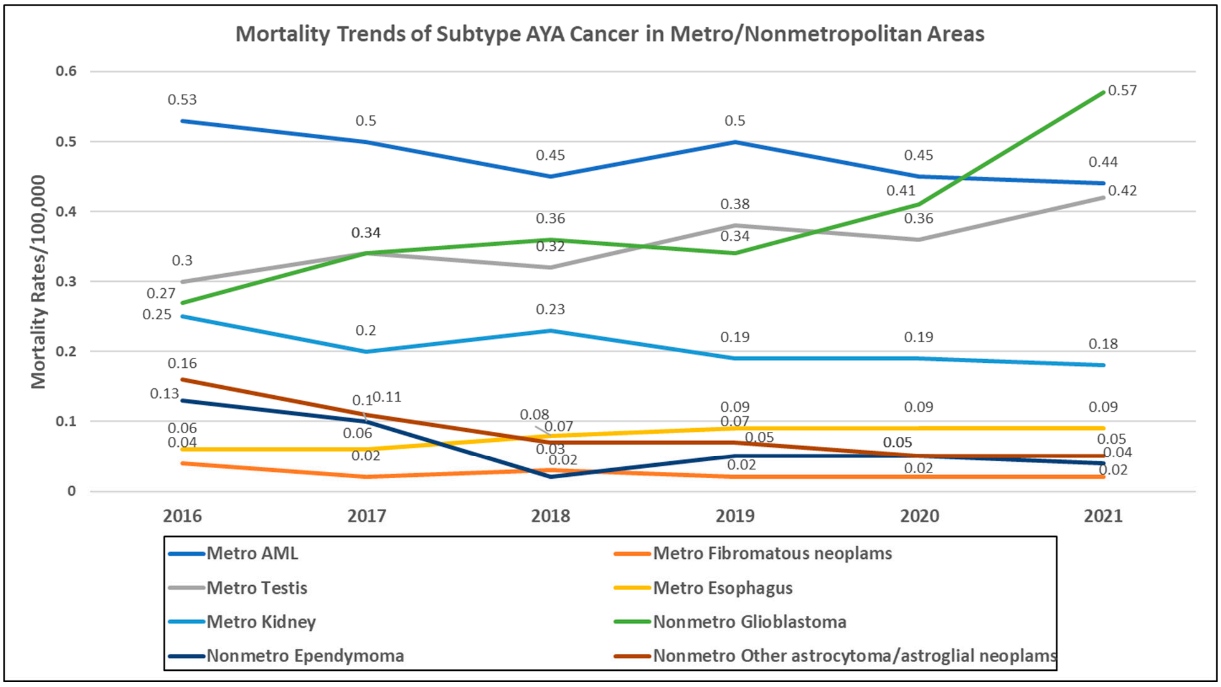

Figure S4. Mortality trends of subtype cancers by metro/nonmetropolitan status when considering multiple primary cancer deaths (significant change of trends)
